# Objectively Measured Physical Activity Is Lower in Individuals with Normal Weight Obesity in the United States

**DOI:** 10.3390/ijerph191811747

**Published:** 2022-09-17

**Authors:** Nadeeja N. Wijayatunga, Heontae Kim, Harry M. Hays, Minsoo Kang

**Affiliations:** 1 Department of Nutrition and Hospitality Management, University of Mississippi, University, MS 38677, USA; 2 Institute of Child Nutrition, University of Mississippi, University, MS 38677, USA; 3 Department of Health, Exercise Science and Recreation Management, University of Mississippi, University, MS 38677, USA

**Keywords:** obesity phenotypes, unhealthy lean, exercise

## Abstract

The role of physical activity in normal weight obesity (NWO), which is associated with increased cardiometabolic risk, is not clear. This study aimed to determine body composition phenotype-specific differences in objectively measured physical activity and sedentary time in adults in the United States. A total of 2055 adults with a body mass index (BMI) ≥ 18.5 m^2^ were studied using 2003–2006 National Health and Nutrition Examination Surveys. Physical activity and percent body fat (BF%) were measured using accelerometer and dual-energy X-ray absorptiometry, respectively. A BF% > 23.1% and >33.3% for men and women, respectively, was considered excess. A BMI of 18.5–24.9 kg/m^2^ with excess BF% was defined as NWO, while those with normal BF%, as normal weight lean (NWL). A BMI of ≥25 kg/m^2^ with excess BF% was considered overweight/obesity (OB). Compared to NWL, moderate to vigorous physical activity was significantly lower by 8.3 min (95% confidence interval/CI = −15.20, −1.40) and 10.18 min (95% CI = −14.83, −5.54) per day in NWO and OB, respectively. Low-intensity physical activity was also significantly lower by 17.71 min (95% CI = −30.61, −4.81) per day in NWO compared to NWL. However, sedentary time was not different. Objectively measured physical activity is significantly lower in NWO compared to NWL, while sedentary time is not.

## 1. Introduction

Body mass index (BMI) is a mathematical formula that is commonly used to screen for obesity and cardiometabolic disease. Because BMI has low sensitivity and specificity, individuals with a normal BMI but high fat are misclassified as “normal weight” [1,2,3]. Normal weight obesity (NWO) syndrome was first described by De Lorenzo et al. as excess adiposity with a normal BMI, higher inflammation, oxidative stress, and altered metabolism compared to those with a normal BMI and normal body fat percentage (normal weight lean/NWL) [2,4]. Furthermore, individuals with NWO may have lower muscle mass, and may have an increased risk for sarcopenia [1,4]. It is estimated that about 30 million Americans have NWO [5], and the prevalence of NWO ranges from 4.5% to 22% among adults in the world [4].

The individuals with NWO have several cardiometabolic abnormalities, such as subclinical vascular inflammation, atherosclerosis, insulin resistance, and impairment of left ventricular function compared to NWL [4,6]. Moreover, the prevalence of high blood pressure, dyslipidemia, and metabolic syndrome is higher in NWO compared to NWL in the United States (USA) when a body fat percent of more than 23.1% and 33.3% is used to define NWO in men and women, respectively [5]. In addition, women with NWO in the USA have a 2.2-fold higher risk for cardiovascular mortality compared to NWL [5]. However, most of the individuals with NWO and their healthcare providers are likely to be unaware of the increased health risks since they are often categorized as “normal weight”.

Physical activity is an important lifestyle behavior for weight control, and to reduce cardiovascular disease (CVD) morbidity and mortality [7]. Physical activity is known to reduce adiposity, improve cardiorespiratory fitness, and reduce overall mortality [8]. Even though the exact etiology of NWO is not clear, reduced physical activity is among some of the possible factors associated with NWO [1,4]. The role of physical activity in individuals with NWO is not clear due to conflicting findings in the past literature [9,10,11]. However, self-reported physical activity energy expenditure is often inaccurate due to over-reporting, difficulty in recalling different types of physical activities, and difficulty in categorizing into different physical activity levels. Even though objectively measured physical activity can provide a more accurate physical activity energy expenditure than self-reported physical activity [12], there is a gap in the literature regarding differences in objectively measured physical activity levels in NWO. The prevalence of NWO (10.8%) is higher among those who are sedentary compared to those who are active (5.3%) among normal-weight Brazilian young adults [10]. However, past studies have not explored the differences between the objective measures of sedentary time between NWL and NWO.

The present study aimed to determine the differences in moderate–to–vigorous physical activity (MVPA) and low-intensity physical activity (LIPA), and sedentary time in adults with different body composition phenotypes including NWL, NWO, and overweight/obesity with high percent body fat (OB) in the USA. We hypothesized that the MVPA and LIPA would be lower while sedentary time would be higher in both the NWO and OB phenotypes compared to NWL. To test our hypotheses, a secondary data analysis was performed using the data from the National Health and Nutrition Examination Survey (NHANES) 2003–2006 cycles.

## 2. Methods

### 2.1. Participants and Study Design

A cross-sectional, secondary data analysis was performed using NHANES 2003–2006 data. This study was conducted by the National Center for Health Statistics as a multistage, stratified, clustered probability design cross-sectional study, and recruited a sample of the civilian non-institutionalized representatives of the population in the USA [13].

Briefly, the participants provided informed consent for the study. The initial interview was performed at home. About 2 weeks later, the participants underwent a standardized medical examination and laboratory testing at a mobile examination unit conducted by trained staff using standardized protocols [13].

A total of 20,470 individuals participated in the NHANES 2003–2006 cycles. For the present study, the inclusion criteria were: (1) age > 19 years, (2) BMI ≥ 18.5 m^2^, and (3) wore the accelerometer for a minimum of 4 valid wear days (which included at least 1 weekend day). The exclusion criteria for this study were: (1) on medication for hypertension (BPQ040A) (*n* = 2780), (2) on insulin (DIQ050) or taking medication for diabetes (DIQ070) (*n* = 633), (3) cancer other than non-melanoma skin cancer (MCQ230A, MCQ230B, MCQ230C, and MCQ230D) (*n* = 757), (4) positive lab pregnancy test or self-reported pregnant at exam (RIDEXPRG) (*n* = 674), and (5) diagnosed with arthritis (MCQ160A) (*n* = 2655). Thus, a total of 2055 individuals were considered as the final sample for the present study.

### 2.2. Procedures

Demographic data on age (RIDAGEYR), sex (RIAGENDR), household income (INDHHINC), education (DMDEDUC2), and race/ethnicity (RIDRETH1) were included. Smoking status was categorized as either never smoked, current smoker, or former smoker based on the answers to the questions regarding smoking 100 cigarettes in a lifetime (SMQ020) and current smoking status (SMQ040). Alcohol consumption was categorized as either never drink, current drinker, or former drinker based on answers to questions regarding having had at least 12 drinks in the past year (ALQ101) or drinking more than 12 drinks in a lifetime (ALQ110).

Weight and height were measured using a digital weighing scale and a fixed stadiometer, respectively, following standard protocols [14,15]. BMI was calculated as weight (kg) divided by squared height in meters (m^2^). The percent body fat was determined using Dual-Energy X-ray Absorptiometry (DXA) (Hologic, Inc., Bedford, MA, USA), using standard protocols. Further details of the anthropometric and body composition measurement methods are documented in the Body Composition Procedures Manual located on the NHANES website [16,17].

Daily physical activity was measured in one-minute intervals for 7 consecutive days using a uniaxial ActiGraph AM-7164 accelerometer (ActiGraph, Ft. Walton Beach, FL, USA) that was worn over the right hip. The participants wore the accelerometer from morning until bedtime and were asked to remove it before bathing or swimming, since it was not waterproof, or if going to sleep. Participants using wheelchairs, or with any disability that prevented them from walking or wearing the accelerometer, were not provided with an accelerometer. Details of physical activity measurements are described on the NHANES website [18,19].

Participants were categorized into three body composition phenotype groups based on BMI (kg/m^2^) and percent body fat measurements from the DXA scan. NWL was defined as normal BMI (18.5–24.9 kg/m^2^) and percent body fat ≤ 23.1% for men and ≤33.3% for women. NWO was defined as normal BMI (18.5–24.9 kg/m^2^) and percent body fat >23.1% and >33.3% for men and women, respectively [5]. The overweight/obesity (OB) phenotype group had a BMI of ≥25 kg/m^2^ with a percent body fat > 23.1% and >33.3% for men and women, respectively.

Physical activity categories and sedentary time were designed as follows. The presence of ≥60 min of consecutive zero counts was considered non-wear time, as described by Troiano et al. [20]. To be considered as a valid day, a participant had to have at least 10 h of wear time. For the present study, only the participants with at least 4 valid days including at least one valid weekend day were included. The Freedson thresholds were used to define different physical activity levels. Amounts of 101–1951 counts/min, 1952–5724 counts/min, and ≥5725 counts/min were the definitions for low intensity, moderate intensity, and vigorous intensity physical activity, respectively [21]. MVPA per day was calculated by the addition of moderate and vigorous physical activity. Time periods with <100 counts/minute were considered as sedentary time and reported as sedentary time per day [21]. LIPA was calculated by deducting the total sedentary time and MVPA time from the total wearing time.

### 2.3. Statistical Analyses

SURVEY procedures in SAS version 9.4 (SAS institute Inc., Cary, NC, USA) were used to perform all statistical analyses. Sample weights, stratification, and clustering in the NHANES data were performed. Any participants with missing data were excluded from the analysis. Participants’ descriptive characteristics by body composition phenotype groups were analyzed as a mean and percentage using SURVEYMEANS and SURVEYFREQ procedures, respectively. Group differences in age and BMI were assessed using one-way ANOVA with Bonferroni correction. Group differences for other categorical variables were assessed using Pearson’s Chi-square test. Differences in LIPA, MVPA, and sedentary time among body composition phenotype groups were measured using multiple linear regression analysis adjusting for age, sex, race/ethnicity, education, income, smoking status, alcohol consumption, and NHANES cycles. Statistical significance was set a priori at *p* value < 0.05.

## 3. Results

The NHANES 2003–2006 cycles contained a total of 20,470 adults over the age of 19 years. However, only 12,662 had data available for objectively measured physical activity and we studied 2055 adults (>19 years) with valid accelerometer data. The study participant characteristics are presented in Table 1. There were significant differences among the groups for both age and BMI (*p* < 0.001). Post hoc analyses showed that significant differences in age and BMI between the groups were found except for the age difference between the NWO and OB groups (*p* = 0.828). Adults in the NWO and OB groups were significantly older and had a higher BMI than NWL (*p* < 0.001).

The differences in physical activity levels and sedentary time between different body composition phenotypes were studied, as shown in Table 2, using multiple linear regression analysis. Compared to the NWL group, the MVPA level was significantly lower by 8.3 (95% CI: −15.20, −1.40) and 10.18 (95% CI: −14.83, −5.54) minutes per day in the NWO and OB groups, respectively, after adjusting for age, sex, race, education, income, smoking, alcohol consumption, and NHANES cycles. LIPA was significantly lower by about 17.71 (95% CI: −30.61, −4.81) minutes per day only in the NWO group compared to NWL after adjusting for age, sex, race, education, income, smoking, alcohol consumption, and NHANES cycles. Compared to NWL, adults in the NWO and OB groups had 7.19 (95% CI: −8.31, 22.70) and 12.07 (95% CI: −3.46, 27.60) more minutes per day of sedentary behavior after adjusting for age, sex, race, education, income, smoking, alcohol consumption, and NHANES cycles, respectively. However, sedentary times were not significantly different for the NWO and OB groups compared to the NWL group.

## 4. Discussion

Overall physical activity and MVPA duration are lower in individuals with overweight and obesity compared to those with a normal weight [22]. However, differences in physical activity and sedentary time in NWO were not studied previously using objective measures. Thus, a cross-sectional analysis was performed using a representative sample of adults in the USA using data from the NHANES 2003–2006 cycles. Differences in accelerometer-based physical activity and sedentary time between body composition phenotypes (NWL, NWO, and OB) in adults in the USA were explored. The main study findings were that individuals with NWO engaged in MVPA and LIPA for a lesser time duration compared to NWL. However, sedentary time was not significantly different in individuals with NWO compared to NWL.

According to the past literature, self-reported physical activity and leisure-time physical activity durations were less in both adults and adolescents with NWO compared to NWL [9,23]. However, not all of the past studies with self-reported physical activity data showed similar findings [11], and this could be due to the inherent inaccuracies associated with self-reported physical activity data [12]. Using objectively measured physical activity data, the present study confirms that individuals with NWO are engaged in less MVPA by about 8 min per day compared to NWL. Regular exercising for more than 20 min per day more than three times per week was less likely to be observed in individuals with NWO according to another study with self-reported physical activity data [24]. However, the present study, with objectively measured physical activity data, suggests that individuals with NWO were engaged in MVPA for about 39 min per day, which adds up to about 276 min of MVPA per week, assuming that they were engaged in the same amount of activity 7 days per week. The American College of Sports Medicine (ACSM) recommends at least 150 to 300 min of moderate intensity aerobic activity, or 75 to 150 min of vigorous intensity activity, and at least two days of resistance training per week for adults [25]. Thus, NWO individuals may require longer durations of MVPA since they have a higher body fat percentage, which should be explored by future studies. However, the presence of longer durations of MVPA in these body composition groups could be because “healthy” participants without hypertension, diabetes, cancer, and arthritis were selected for this analysis.

According to the present study, the individuals with NWO had a significantly lower engagement even in LIPA by about 17 min per day compared to NWL, but sedentary time was not significantly different. While this seems like a modest decrease in activity, the implications for health could be drastic. According to a systematic review and meta-analysis, engagement in LIPA can improve cardiometabolic health and lower mortality. Engagement in LIPA for more than 150 min per day in programs that lasted 12 weeks or more also resulted in a reduction in body fat, blood pressure, and blood lipids [26]. Future studies should explore the role of LIPA in NWO.

Sedentary time is associated with obesity, and increased cardiovascular morbidity and mortality [27,28,29,30]. Sedentary time positively correlates with increases in waist circumference and clustered metabolic risk scores independent of MVPA [31,32]. Even though both NWO and OB groups had higher sedentary time than NWL, it was not significantly different in the present study, which could be because only “healthy” adults were selected.

Exercise and health benefits have a dose–response association [33]. Decreases in physical activity/fitness may increase the risk for CVD, metabolic syndrome, type II diabetes, non-alcoholic fatty liver disease, some cancers, and overall mortality, independent of weight loss [34,35]. Ten weeks of circuit resistance training reduced body fat and fasting glucose but increased lean mass, cardiovascular fitness, muscle strength, and endurance in NWO [36]. Based on the present study findings, adults with NWO may need to increase engagement in all types of physical activities to increase MVPA and LIPA.

Maffetone et al. suggested that “normal weight” status is a barrier for individuals with NWO to be motivated to engage in healthy behaviors [37]. The Health Belief Model is a social cognitive model that helps to explain engagement in health behaviors by an individual. Perceived severity and susceptibility are some of the constructs included in this model [38]. It may be that perceived susceptibility for disease and perceived severity of obesity is not clear to the individuals with NWO. Thus, a detailed analysis of the psychological determinants of physical activity in NWO is warranted in future studies, though it is beyond the scope of the present study.

The findings from this study have a clinical application. Since both MVPA and LIPA are lower in NWO, such individuals should be encouraged to be more physically active. Relative thinness and “normal weight” status based on BMI classification may overshadow the health risks associated with increased body fat, and could be a barrier for individuals to engage in healthy behaviors [37]. In individuals with obesity, the diagnosis of obesity is associated with at least 5% weight loss [39]. Hence, increasing perceived susceptibility and severity by informing those individuals that they have excess adiposity, and an explanation of their increased health risks by their healthcare providers may motivate them to engage in healthy behaviors. The findings from this study will also be important to develop behavioral interventions to improve health in NWO.

The present study has several strengths. NHANES data, which is a large dataset that is representative of the population in the USA, were used for this study. Unlike previous studies, objectively measured physical activity data were obtained from an accelerometer. In addition, we studied relatively healthy adults to avoid any confounding effects that a cardiometabolic disease can have on motivation for physical activity, since an individual with such conditions may be advised to exercise. However, the present study has a few limitations. In the absence of a standard definition for NWO, we used body fat percent cutoffs of more than 23.1% and 33.3% for men and women, respectively, to define NWO, because Romero-Corral et al. reported that NWO based on these cutoffs is associated with increased cardiovascular risk prevalence in adults in the USA [5]. This study is a cross-sectional study and that limits the ability to determine causations; only associations can be determined. The accurate quantification of exercise on a stationary bike, row machines, certain sports, or on elliptical trainers is limited due to the use of a uniaxial accelerometer in the NHANES study [40,41]. Since the data were collected in 2003–2006, the physical activity levels may be different to present times.

## 5. Conclusions

In summary, healthy USA adults with NWO and overweight/obesity engaged in significantly less MVPA compared to NWL. Healthy adults with NWO also engaged in significantly less LIPA compared to NWL. However, sedentary time was not significantly different between body composition groups. The findings from this study suggest that individuals with NWO may need to perform more physical activity. This highlights the importance of measuring body fat percentage to identify NWO during routine health screenings.

## Figures and Tables

**Table 1:** Study participant characteristics.

9		All (*n* = 2055)	NWL (*n* = 363)	NOW (*n* = 387)	OB (*n* = 1305)	*p*-Value
Age (years)		39.39 ± 0.44	34.57 ± 0.67 ^a^	40.48 ± 1.01 ^b^	40.71 ± 0.43 ^b^	<0.001
BMI (kg/m^2^)		27.27 ± 0.17	21.87 ± 0.08 ^a^	23.19 ± 0.06 ^b^	30.42 ± 0.18 ^c^	<0.001
Sex	Male	1147 (54.12%)	205 (50.67%)	182 (45.10%)	760 (58.15%)	0.003
	Female	908 (45.88%)	158 (49.33%)	205 (54.90%)	545 (41.85%)	
Race	Mexican American	510 (9.82%)	59 (6.42%)	86 (8.56%)	365 (11.38%)	<0.001
	Other Hispanic	78 (3.99%)	12 (3.69%)	14 (3.49%)	52 (4.25%)	
	Non-Hispanic White	1034 (70.49%)	207 (73.87%)	220 (73.88%)	607 (68.25%)	
	Non-Hispanic Black	350 (10.27%)	70 (10.98%)	39 (4.95%)	241 (11.71%)	
	Other	83 (5.44%)	15 (5.04%)	28 (9.11%)	40 (4.41%)	
Education	≤Some high school	456 (12.44%)	70 (11.93%)	78 (11.27%)	308 (12.98%)	0.008
	High School Graduate/GED or Equivalent	472 (23.36%)	62 (16.36%)	103 (24.26%)	307 (25.48%)	
	Some college/Associate’s degree	632 (34.28%)	122 (35.74%)	104 (30.72%)	406 (34.90%)	
	≥College Graduate	495 (29.93%)	109 (35.98%)	102 (33.74%)	284 (26.64%)	
Income	<USD 25,000	499 (17.37%)	102 (21.91%)	98 (17.69%)	299 (15.71%)	0.429
	USD 25,000 to <45,000	493 (21.67%)	82 (21.69%)	91 (20.54%)	320 (22.03%)	
	USD 45,000 to <75,000	491 (26.32%)	82 (24.37%)	91 (26.80%)	318 (26.84%)	
	≥USD 75,000	572 (34.63%)	97 (32.03%)	107 (34.97%)	368 (35.42%)	
Smoking	Never smoker	1139 (55.90%)	193 (56.72%)	200 (53.88%)	746 (56.26%)	0.003
	Current smoker	463 (21.37%)	59 (15.50 %)	85 (19.73%)	319 (23.91%)	
	Former smoker	453 (22.73%)	111 (27.78%)	102 (26.39%)	240 (19.83%)	
Alcohol	Lifelong abstainers	238 (10.98%)	41 (10.21%)	53 (13.99 %)	144 (10.29%)	0.002
	Ex-drinkers	320 (13.57%)	35 (7.21%)	66 (14.99%)	219 (15.31%)	
	Current drinkers	1497 (75.45%)	287 (82.59%)	268 (71.02%)	942 (74.40%)	

Mean (standard error) is shown for continuous data, and a one-way ANOVA with post hoc analysis and Bonferroni correction was performed. ^a, b, c^ Significant differences between different letters; no differences between the same letters. Frequency (percent) is shown for categorical data, and Pearson Chi-square test *p* values are shown for continuous and categorical variables, respectively. Abbreviations: BMI = Body Mass Index; GED = General Educational Development; NWL = Normal Weight Lean; NWO = Normal Weight Obesity; OB = Overweight/Obesity.

**Table 2:** Differences in physical activity and sedentary time (minutes per day) between the body composition groups.

	Without Adjustment		Model 1	
	Estimate	95% CI	Estimate	95% CI
**Moderate to vigorous PA**				
Intercept	42.32	37.35, 47.28	47.68	32.95, 62.41
NWO	−11.84	−18.64, −5.05	−8.30	−15.20, −1.40
OB	−11.25	−15.91, −6.59	−10.18	−14.83, −5.54
**Light intensity PA**				
Intercept	367.50	355.12, 379.87	376.34	319.12, 433.56
NWO	−16.68	−29.69, −3.66	−17.71	−30.61, −4.81
OB	−4.59	−19.03, 9.86	−10.44	−25.27, 4.40
**Sedentary time**				
Intercept	463.46	451.14, 475.79	355.91	301.97, 409.85
NWO	9.60	−5.51, 24.71	7.19	−8.31, 22.70
OB	12.23	−2.44, 26.90	12.07	−3.46, 27.60

Linear regression analysis was performed and the NWL group was used as the reference group. Model 1 was adjusted for age, sex, race, education, income, smoking, alcohol consumption, and NHANES cycles. Abbreviations: NWL = Normal Weight Lean; NWO = Normal Weight Obesity; OB = Overweight/Obesity; PA = Physical Activity.

## Data Availability

All data analysis were performed using publicly available data which can be accessed at: https://wwwn.cdc.gov/nchs/nhanes/continuousnhanes/default.aspx?BeginYear=2003 and https://wwwn.cdc.gov/nchs/nhanes/continuousnhanes/default.aspx?BeginYear=2005 (accessed on 23 March 2021).
